# Magnetic resonance imaging evaluation of meniscoid superior labrum: normal variant or superior labral tear*

**DOI:** 10.1590/0100-3984.2015.0083

**Published:** 2016

**Authors:** Marcelo Novelino Simão, Emily N. Vinson, Charles E. Spritzer

**Affiliations:** 1 MD, PhD, Radiologist at the Central de Diagnóstico Ribeirão Preto (Cedirp), Attending Physician in the Musculoskeletal Division of the Center for Imaging at the Hospital das Clínicas da Faculdade de Medicina de Ribeirão Preto da Universidade de São Paulo (HCFMRP-USP), Ribeirão Preto, SP, Brazil.; 2 MD, Assistant Professor of Radiology, Duke University, Durham, NC, USA.; 3 MD, Chief of the Musculoskeletal Imaging Division, Duke University, Durham, NC, USA.

**Keywords:** Magnetic resonance imaging, Shoulder/injuries, Shoulder joint/physiopathology, Fibrocartilage/labrum

## Abstract

**Objective:**

The objective of this study was to determine the incidence of a "meniscoid" superior labrum.

**Materials and Methods:**

This was a retrospective analysis of 582 magnetic resonance imaging examinations of shoulders. Of those 582 examinations, 110 were excluded, for a variety of reasons, and the final analysis therefore included 472 cases. Consensus readings were performed by three musculoskeletal radiologists using specific criteria to diagnose meniscoid labra.

**Results:**

A meniscoid superior labrum was identified in 48 (10.2%) of the 472 cases evaluated. Arthroscopic proof was available in 21 cases (43.8%). In 10 (47.6%) of those 21 cases, the operative report did not include the mention a superior labral tear, thus suggesting the presence of a meniscoid labrum. In only one of those cases were there specific comments about a mobile superior labrum (i.e., meniscoid labrum). In the remaining 11 (52.4%), surgical correlation demonstrated superior labral tears.

**Conclusion:**

A meniscoid superior labrum is not an infrequent finding. Depending upon assumptions and the requirement of surgical proof, the prevalence of a meniscoid superior labrum in this study was between 2.1% (surgically proven) and 4.8% (projected). However, superior labral tears are just as common and are often confused with meniscoid labra.

## Abstract (pt)

**Objetivo:**

O objetivo deste estudo é determinar a incidência do lábio superior da glenoide com aspecto meniscoide.

**Materiais e Métodos:**

Foi feita análise retrospectiva de 582 exames de ressonância magnética do ombro. Foram excluídos 110 casos por motivos diversos, restando 472 casos para avaliação. A leitura foi feita em consenso por três médicos radiologistas musculoesqueléticos.

**Resultados:**

Lábio superior meniscoide foi encontrado em 48 casos (10,2%). Resultados de artroscopia estavam disponíveis em 21 casos (43,8%). Em 10 destes casos (47,6%) não havia informação de lesão labral superior, sugerindo a presença de lábio meniscoide. Em apenas um caso havia comentário sobre um lábio superior hipermóvel, indicando um lábio meniscoide. Nos 11 casos restantes (52,4%) a correlação cirúrgica demonstrou lesão do lábio superior.

**Conclusão:**

Lábio superior de aspecto meniscoide não é um achado infrequente. Dependendo das hipóteses assumidas e da necessidade de prova cirúrgica, a prevalência de um lábio superior meniscoide varia entre 2,1% (comprovação cirúrgica) e 4,8% (projeção) nesta série. Entretanto, as lesões labrais superiores têm uma prevalência parecida e podem ser confundidas com o aspecto de um lábio meniscoide.

## INTRODUCTION

The clinical diagnosis of superior glenoid labrum pathology is difficult^(1,2)^ and may be confused with or accompanied by impingement syndrome, rotator cuff tears, or other shoulder pathologies. Magnetic resonance imaging (MRI) of the superior labrum is an accepted method for diagnosing labral tears. However, for maximum accuracy, the radiologist must be aware of a number of normal superior labral variants that can hinder correct interpretation. Sublabral foramen, Buford complex, cord-like middle glenohumeral ligament, sublabral recess, and articular cartilage interface are well-known normal anatomical variants, all of which are well described in the radiology literature^(3-7)^.

Albeit well recognized by orthopedic surgeons^(1,8-12)^, a meniscoid superior labrum has received little attention in the radiology literature^3,13-15^. A meniscoid superior labrum should be considered when the free edge of the labrum drapes over the underlying glenoid at the 12 o'clock position. A meniscoid labrum is mobile because the base of the labrum is not firmly attached to the superior glenoid near the anchor of the long head of the biceps. That type of attachment results in a superior sublabral recess^(13)^. Although this recess is easily recognized when there is intra-articular fluid interposed between the labrum and the glenoid, secondary to an effusion or MR arthrography, the recess may be much more difficult to identify when there is no intra-articular fluid. Therefore, a meniscoid appearance to the superior labrum may be the only finding.

The purpose of this study was to determine the prevalence of a meniscoid superior labrum, as defined in the orthopedic literature (labral coverage of the superior glenoid cartilage), in a large sample of MRI scans performed for multiple reasons.

## MATERIALS AND METHODS

### Study population

Institutional review board approval and waiver of informed consent were obtained prior to the start of this retrospective study. Over a seven-month period, 582 consecutive shoulder MRI examinations were performed at our institution. Of those, 110 were excluded, for the following reasons: patient below 18 years of age (*n* = 20); nonstandard protocol (*n* = 15); postoperative shoulder study (*n* = 59); significant artifacts (*n* = 3); and miscellaneous (*n* = 12). The remaining 472 examinations were evaluated as described below.

Of the 472 patients in the studied population, 250 (53%) were male and 222 (47%) were female. The mean age was 52.9 years (range, 18-86 years).

### MRI technique

Patients included in this study underwent MRI in a 1.5 T scanner (Signa; GE Healthcare, Milwaukee, WI, USA, and Avanto; Siemens Medical Solutions, Erlangen, Germany) or in a 3.0 T scanner (HDx; GE Healthcare, and Trio; Siemens Medical Solutions). Approximately three quarters (76.9%) of the scans were obtained at 1.5 T, and approximately one quarter (23.1%) were obtained at 3.0 T. All scans were obtained in accordance with our conventional shoulder MRI protocol or our shoulder MR arthrography protocol. Approximately three quarters of the studies (76.6%) involved standard MRI, and approximately one quarter (23.4%) involved MR arthrography. Our conventional shoulder MRI protocol consists of axial and oblique coronal fat-suppressed fast spin-echo (FSE) T2-weighted images [repetition time/echo time (TR/TE), 4000/65-75 ms] fat-suppressed FSE proton density images (TR/TE, 3500-4000/20-35 ms), oblique sagittal T1-weighted images (TR/TE, 500-620/14-15 ms), and oblique sagittal fat-suppressed FSE T2-weighted images (TR/TE, 4000/65-75 ms). The MR arthrography studies were obtained following intra-articular instillation of 0.1 mL of the gadolinium-based contrast agent gadoteridol (ProHance; Bracco Diagnostics, Princeton, NJ, USA) diluted in 12 mL of normal saline. The contrast was administered under fluoroscopic guidance via an anterior approach. Our shoulder MR arthrography protocol included axial, oblique coronal, and oblique sagittal fat-suppressed FSE T2-weighted images (TR/TE, 3000-4000/65-75 ms) and fat-suppressed T1-weighted images (TR/TE, 500-650/14-15 ms), and oblique sagittal T1-weighted images without fat suppression (TR/TE, 500-617/14-15 ms). The number of excitations was 2 for FSE sequences and 2 for T1-weighted sequences; the echo train length was typically 8 for all FSE sequences. In all studies, the slice thickness was 4 mm, and the interslice gap was 0.4 mm, with a 14 cm field of view and a 256 × 192 matrix.

### Image analysis

**Figure 1:**
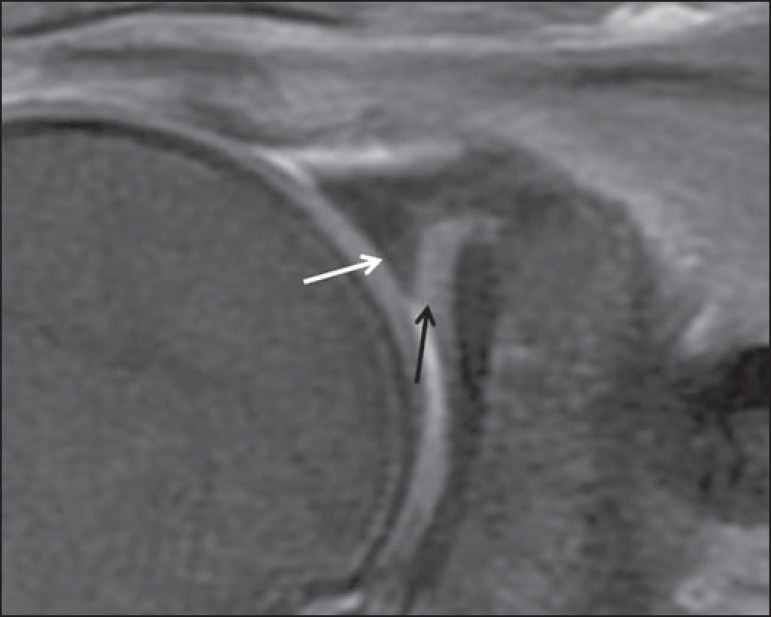
35-year-old male. Coronal T2-weighted MRI scan with fat saturation, showing a prominent superior labrum with a inferior free edge covering a portion of the glenoid articular surface (white arrow), which is described as meniscoid superior labrum. Superior glenoid cartilage (black arrow).

Three radiologists, each with at least eight years of experience in musculoskeletal imaging, evaluated the MR images on a workstation meeting the Digital Imaging and Communications in Medicine standards (Centricity; GE Healthcare). The scans were interpreted by consensus. The superior labrum was investigated for the presence of a meniscoid labrum. A meniscoid superior labrum was defined as a prominent superior labrum with a inferior free edge that covered a portion of the glenoid articular surface (Figure 1). Because we were interested only in the size, shape, and position of the superior labrum, as described above, we did not attempt to conduct a formal analysis to distinguish among a meniscoid labrum, an isolated but prominent sublabral recess, and a superior labral tear.

## RESULTS

The consensus readings identified meniscoid superior labrum in 48 (10.2%) of the 472 cases evaluated. Among the 48 patients involved in those cases, the mean age was 51 years (range, 19-72 years) and males accounted for 34 (71%).

**Figure 2:**
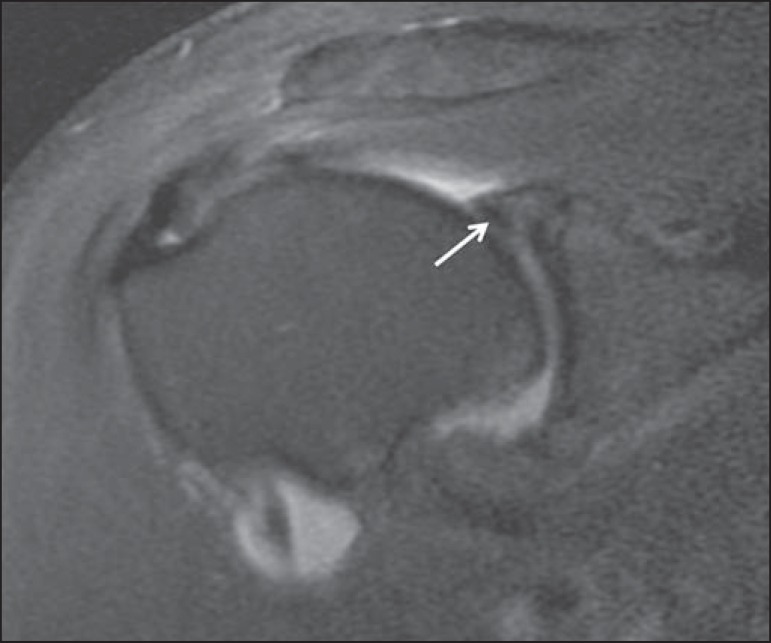
56-year-old male. Coronal T2-weighted MRI scan with fat saturation. The MRI consensus interpretation was meniscoid superior labrum (arrow). The operative report mentioned a mobile superior labrum without a tear, indicating a truly meniscoid normal variant.

**Figure 3:**
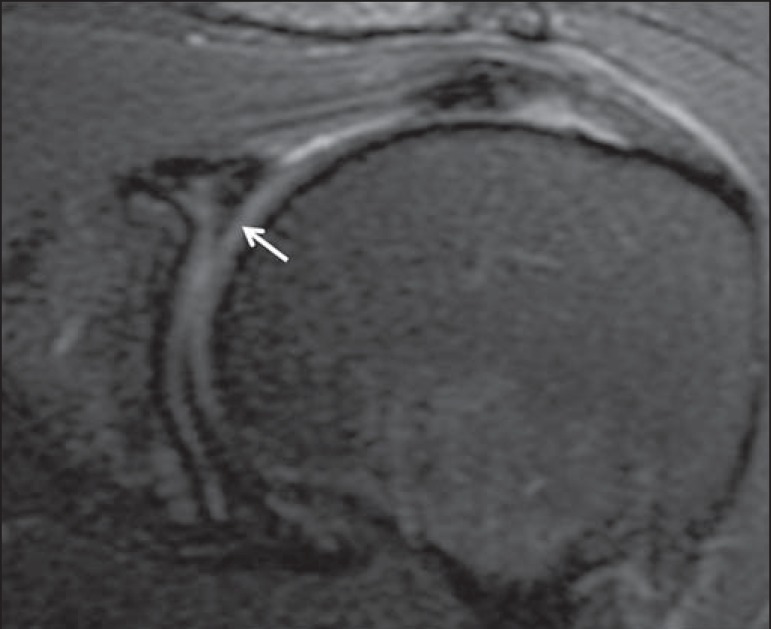
54-year-old female. Coronal T2-weighted MRI scan with fat saturation. The consensus MRI reading was meniscoid superior labrum (arrow). The operative report included no mention of a labral tear, meniscoid superior labrum therefore being presumed to be a normal variant.

**Figure 4:**
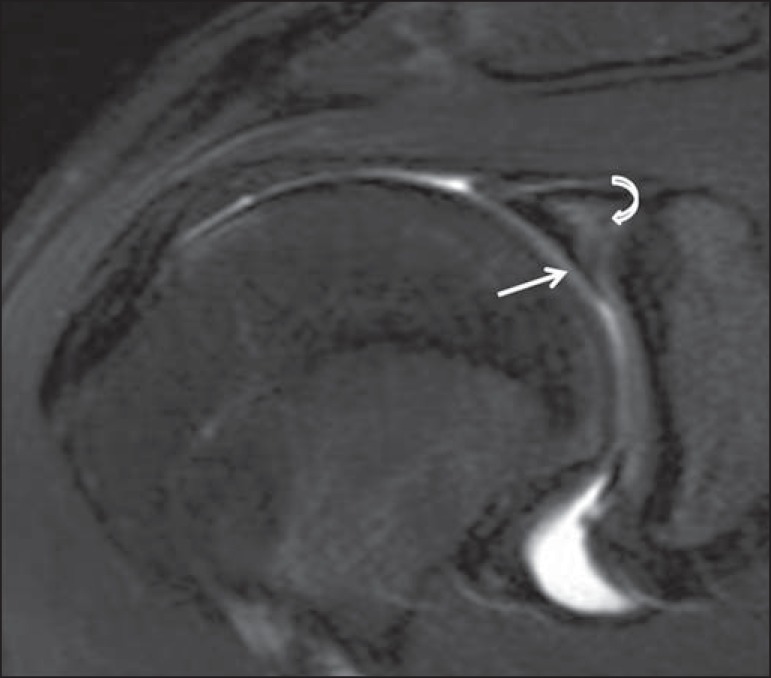
20-year-old male. Coronal T2-weighted MRI scan with fat saturation, showing a meniscoid superior labrum (straight arrow). The operative report mentioned a superior labral tear. Note the typical lateral high signal orientation within the labrum, which is most likely to represent a superior labral tear (curved arrow).

Arthroscopic correlation was available in 21 (43.8%) of the 48 cases. In 10 (47.6%) of those 21 cases, no superior labral tear was mentioned in the operative report. A labrum with a mobile free edge without a tear, indicative of a meniscoid superior labrum (Figure 2), was specifically mentioned in only one operative note. In the remaining 11 cases (52.4%), the reports contained no mention of a superior labral tear was mentioned in the reports, although they also contained no other descriptive comments about the labra. Those labra were assumed to be intact and as such to meet the criteria for meniscoid labra (Figure 3). However, a superior labral tear was subsequently identified in all 11 of those cases: in 7, the tear was restricted to the superior labrum, indicating a typical superior labral anterior posterior (SLAP) tear; in the remaining 4 cases, the superior labral tear was part of a more extensive labral tear extending anteriorly or posteriorly (Figure 4).

Depending upon assumptions and the requirement of surgical proof, the prevalence of a meniscoid superior labrum was between 2.1% (if we counted only the 10 cases of surgically confirmed normal superior labra) and 7.8% (if we assumed that the 27 meniscoid labra without surgical proof were all normal). However, if we assume the same proportions of normal and torn labra in the remaining 27 meniscoid superior labra without surgical confirmation, a prevalence of 4.8% is projected.

## DISCUSSION

Recent studies conducted in Brazil have emphasized the role of MRI in evaluating the musculoskeletal system, especially the shoulder^(16-19)^. The labrum is a fibrocartilaginous structure that occupies the transition zone between the underlying articular cartilage and the fibrous tissue of the joint capsule. The morphology of the labrum differs between its superior and inferior portions^(20)^.

Shoulder pain can be related to subacromial impingement, acromioclavicular degenerative disease, glenoid labral pathology, or a variety of other derangements. Although clinical tests may suggest a diagnosis of labral tear, their sensitivity and specificity are relatively low^(2,20)^. Various methods, such as ultrasound and computed tomography arthrography, have been tested as means of evaluating labral anatomy and pathology^(21-23)^. However, MRI and MR arthrography are considered the most reliable methods for diagnosing superior labral anatomy and pathology^(14,15,24)^. In a meta-analysis of the available literature on the topic, Smith et al.^(25)^ concluded that MR arthrography is only marginally superior to conventional MRI for the detection of glenohumeral labral lesions. In addition, the authors found that the reported diagnostic accuracy was greatest in the studies involving the use of 3.0 T MRI scanners. However, diagnosing superior labral pathology with MRI is not without problems. Some authors have suggested that conventional MRI is not suitable for diagnosing SLAP tears, because, despite a high level of sensitivity, it has low specificity^(26)^. The difficulty in discriminating normal variations from labral tears is emphasized in the radiology literature^(4-6,13,14,27,28)^. Such difficulty is especially pronounced when one attempts to distinguish a sublabral recess from a superior labral or SLAP tear, because all three occur superiorly and because sublabral recess has features in common with a superior labral tear. In a review article about SLAP lesions, Chang et al.^(5)^ discussed several normal variants, including the superior labral recess, but did not mention a meniscoid superior labrum. It is our opinion that knowledge of a meniscoid variant would help improve MRI assessment of the superior labrum.

A handful of articles in the orthopedic literature describe meniscoid superior labrum as a normal variant^(1,8,11-13)^, although it could also be secondary to a SLAP tear^(14)^. The superior labrum is typically described as triangular but may be meniscoid in shape if the inner (inferior) free edge partially covers the glenoid articular surface^(11)^. Other authors have described some superior labra as meniscal in appearance and loosely attached to the underlying glenoid^(1,9,10,20)^. It has also been postulated that a nonpathologic meniscoid superior labrum is mobile and is associated with smooth cartilage on the supraglenoid tubercle^(1)^.

Davidson et al.^(1)^ identified mobile superior labra in a subgroup of 49 patients within a sample of 191 consecutive patients undergoing arthroscopy. The authors described three types of labrum: triangular (not draped over the glenoid surface), in 44%; meniscoid (partially extending over the glenoid articular surface), in 38%; and "bumper" (characterized by a small excrescence of fibrous tissue, which probably represents a more prominent meniscoid labrum), in 18%. Lee et al.^(29)^ stated that the superior third of the labrum may resemble a knee meniscus and described a case of a labrum variant covering nearly all of the glenoid surface, the exception being a small central area. The authors suggested that this incomplete discoid labrum was caused by excessive superior loading of the labrum, given that the patient was in a wheelchair.

In the radiology literature, a meniscoid superior labrum has received little attention. On MRI scans, the labral morphology is typically described as triangular but may also be round, crescent-shaped, or blunted. A superior recess is considered the most common normal variation, occurring in up to 73% of patients. A mobile superior labrum, with no reference to a meniscoid appearance, has been reported to occur in up to 25% of individuals^(7)^. However, a meniscoid shape is rarely mentioned in the literature. Kwak et al.^(3)^ defined type III biceps-labral complex as a condition in which the superior labrum is shaped like a meniscus and there is a large sublabral sulcus that projects under the labrum and over the cartilaginous portion of the glenoid. In a review of MR arthrograms of 80 patients who also underwent arthroscopy, Jee et al.^(14)^ implicated meniscoid-type superior labrum as a cause of a false-positive interpretation in superior labral tears, although they described a meniscoid superior labrum at surgery in only one patient, and that labrum was accompanied by a type II SLAP tear. In a prospective study of 104 indirect MR arthrograms with arthroscopic correlation, Dinauer et al.^(15)^ described a case of frayed meniscoid superior labrum misinterpreted as a type II SLAP tear.

To our knowledge, the only radiological study of meniscoid superior labra was conducted by Manvar et al.^(13)^, who used MRI in attempting to distinguish them from superior labral tears. The authors considered a meniscoid superior labrum to be part of the superior sublabral recess and stated that the best criterion to distinguish a meniscoid superior labrum from a SLAP tear was the finding of a focal region of abnormal signal beneath the labrum immediately posterior to the biceps anchor without more posterior extension. However, they did not provide sensitivity or specificity data; nor did they discuss the meniscoid appearance of the labrum itself.

Our results indicate a prevalence of meniscoid superior labrum between 2.1% (surgically proven) and 7.8%, with a projected occurrence of 4.8%. This is similar to the 6% incidence reported by Davidson et al.^(1)^ in a study of 191 arthrograms and higher than the prevalence of other wellknown superior labral variations, with the exception of sublabral recess. For example, Buford complex has an approximate prevalence of 1.5%^(3)^.

This study has several limitations. Because it was a retrospective study, it has all of the biases inherent to such a study design. The number of cases with surgical correlation was relatively small, and the surgeons involved did not specifically assess the superior labrum for a meniscoid variant. In only one case did the arthroscopist specifically describe a truly mobile meniscoid superior labrum. Although arthroscopic criteria were adapted for meniscoid labrum detection, specific MRI criteria were not available for assessment. Nonarthrographic MRI is limited in its ability to define the inferior free edge of the labrum, because there is no fluid penetration between the labrum and the glenoid surface. Finally, we did not formally attempt to identify and distinguish among sublabral recesses, sublabral foramina, superior labral tears, and other variations of the superior meniscoid labra. We also did not try to determine whether the cases of meniscoid labrum were related to a prominent sublabral recess, associated with a loose meniscal attachment, as isolated finding, or a part of SLAP tear.

In conclusion, our findings suggest that a meniscoid superior labrum is not an infrequent occurrence. We found the prevalence of a meniscoid superior labrum as a normal variation to be between 2.1% (surgically proven) and 4.8% (as a projected occurrence). Because this variant appears similar to a SLAP tear and we did not attempt to make that differential diagnosis, further studies are needed in order to validate the MRI criteria for meniscoid superior labra and to broaden the distinction between these two entities.
